# Cholesterol binding to ion channels

**DOI:** 10.3389/fphys.2014.00065

**Published:** 2014-02-26

**Authors:** Irena Levitan, Dev K. Singh, Avia Rosenhouse-Dantsker

**Affiliations:** Division of Pulmonary, Critical Care, Sleep, and Allergy, Department of Medicine, University of Illinois at ChicagoChicago, IL, USA

**Keywords:** lipids, cholesterol, membrane proteins, ion channels, cholesterol-binding motifs

## Abstract

Numerous studies demonstrated that membrane cholesterol is a major regulator of ion channel function. The goal of this review is to discuss significant advances that have been recently achieved in elucidating the mechanisms responsible for cholesterol regulation of ion channels. The first major insight that comes from growing number of studies that based on the sterol specificity of cholesterol effects, show that several types of ion channels (nAChR, Kir, BK, TRPV) are regulated by specific sterol-protein interactions. This conclusion is supported by demonstrating direct saturable binding of cholesterol to a bacterial Kir channel. The second major advance in the field is the identification of putative cholesterol binding sites in several types of ion channels. These include sites at locations associated with the well-known cholesterol binding motif CRAC and its reversed form CARC in nAChR, BK, and TRPV, as well as novel cholesterol binding regions in Kir channels. Notably, in the majority of these channels, cholesterol is suggested to interact mainly with hydrophobic residues in non-annular regions of the channels being embedded in between transmembrane protein helices. We also discuss how identification of putative cholesterol binding sites is an essential step to understand the mechanistic basis of cholesterol-induced channel regulation. Clearly, however, these are only the first few steps in obtaining a general understanding of cholesterol-ion channels interactions and their roles in cellular and organ functions.

## Introduction: cholesterol regulation of ion channels

Cholesterol is one of the major lipid components of the plasma membrane of most euakaryotic cells constituting 10–45 mol% with respect to other lipids (Yeagle, 1985, 1991). Normal physiological levels of cholesterol in the plasma membrane are essential to maintain membrane fluidity, thickness, and compartmentalization of the lipid domains that constitute scaffolds for multiple signaling platforms. An increase in membrane cholesterol, however, may underlie cellular and tissue dysfunction and could contribute to the pathological effects of hypercholesterolemia. It is essential, therefore, to understand the mechanisms responsible for cholesterol regulation of membrane proteins. The current review focuses on discussing the evidence for direct cholesterol interactions with ion channels.

Numerous studies showed that a variety of ion channels are sensitive to the level of membrane cholesterol with the most common effect being suppression of channel activity that may include decrease in the open probability, unitary conductance, and/or the number of active channels on the membrane [reviewed by Martens et al. (2004); Maguy et al. (2006); Levitan (2009); Levitan et al. (2010)]. This effect was observed in multiple types of K^+^ channels, including inwardly-rectifying K^+^ channels (Romanenko et al., 2004a), Ca^+2^-sensitive K^+^ channels (Crowley et al., 2003; Bukiya et al., 2011) and voltage-gated K^+^ channels (Hajdú et al., 2003; Abi-Char et al., 2007), as well as in voltage-gated Na^+^ and Ca^+2^ channels (Lundbaek et al., 2004), volume-regulated anion channels (Romanenko et al., 2004b) and vanilloid transient receptor potential channels (TrpV) (Picazo-Juarez et al., 2011). However, cholesterol may also be required for the functional activity of the channels, as it was shown for nicotinic acetylcholine receptor (nAChR) [reviewed by (Barrantes, 2004, 2007) and GABA_*A*_ receptors (Sooksawate and Simmonds, 2001)]. Epithelial Na^+^ channels (eNaC) and several sub-types transient receptor potential (Trp) channels were also shown to be inhibited by the removal of membrane cholesterol [reviewed by Levitan et al. (2010)]. Surprisingly, our recent studies showed that cholesterol may have opposite effects on channel function even within one sub-family of channels (Rosenhouse-Dantsker et al., 2010). In terms of the mechanism, one possibility is that cholesterol interacts directly with a channel protein and regulates its function as a specific ligand. An alternative possibility is that cholesterol may regulate the channels by altering the physical properties of the lipid bilayer which in turn affects protein function. More specifically, it was proposed that cholesterol may regulate ion channels by an increase in bilayer stiffness and hydrophobic mismatch between the transmembrane domains and the lipid bilayer (Lundbaek et al., 1996; Lundbaek and Andersen, 1999). Discrimination between these possibilities is the major challenge in elucidating the mechanisms of cholesterol regulation of any specific type of an ion channel.

Direct interaction between steroids and ion channels was first demonstrated for the nAChR based on the analysis of lipid mobility in the vicinity of the protein (Marsh and Barrantes, 1978). It is important to note, however, that evidence for direct interaction does not necessarily discriminate between the two types of mechanisms described above because cholesterol may still act both, as a ligand or as a modifier of the membrane bilayer in the close vicinity of the channel altering the hydrophobic interaction between the channel and the lipids. This question was further addressed in several studies by altering the sterol composition of the membrane substituting native cholesterol with an array of sterols that have similar effects on the physical properties of the membrane (Popot et al., 1978; Romanenko et al., 2002, 2004b; Addona et al., 2003; Singh et al., 2009; Bukiya et al., 2011 #2464). Furthermore, direct binding between cholesterol and an ion channel has been demonstrated for nAChR using a photoactivatable cholesterol probe (Corbin et al., 1998; Hamouda et al., 2006a) and for a bacterial K^+^ channel using native cholesterol (Singh et al., 2011). Most recently, several studies provided the first insights into the structural determinants of cholesterol-ion channel interactions identifying several structural motifs that are proposed to be responsible for cholesterol binding (Picazo-Juarez et al., 2011; Singh et al., 2012; Rosenhouse-Dantsker et al., 2013).

## Direct association of sterols with nicotinic acetylcholine receptor: biophysical studies

### Lipid belt of immobilized lipids

Early studies of two groups, Barrantes and colleagues and Changeux and colleagues, were first to propose direct interaction of cholesterol with an ion channel based on different experimental approaches. Barrantes and colleagues identified a unique population of lipids that are associated with nAChR and are immobilized at the protein-lipid membrane interface (Marsh and Barrantes, 1978). Specifically, lipid-protein interactions of nAChR were analyzed using electron spin resonance (ESR) spectra of several lipid probes to reveal a population of lipids that are immobilized with respect to the protein and distinct from the general fluid lipid bilayer (Marsh and Barrantes, 1978). This conclusion was based on the detection of two-component ESR spectra for both types of probes with the less mobile component being observed only in the presence of the acetylcholine receptor protein. This effect, however, is not specific for a particular type of lipid and is observed for several lipid species including fatty acids (stearic acid), steroids (androstane), as well as phosphatidylcholine, phosphatidylethanolamine, phosphatic acid, and phosphatidylserine (Marsh and Barrantes, 1978; Marsh et al., 1981; Ellena et al., 1983). Changeux and colleagues used a different approach: they measured the surface pressure of the membrane after injecting the protein into lipid monolayer films prepared from different lipids (Popot et al., 1978). Using both approaches, it was shown that nAChR associates preferably with sterols than with other lipids (Popot et al., 1978; Ellena et al., 1983). In terms of the functional effect of cholesterol on nAChR, multiple studies have shown that incorporation of cholesterol into the phospholipid mixture enhances the functional activity of nAChR in reconstituted lipid vesicles and that nAChR-mediated ion fluxes increase proportionally to the amount of cholesterol in the membrane (up to ~50%) (Dalziel et al., 1980; Criado et al., 1982; Barrantes, 2004, 2007). It was further demonstrated that while the requirement for cholesterol is not absolute, increasing cholesterol level in the membrane stabilizes nAChR channels in the resting state that undergoes agonist-induced activation whereas at low cholesterol level the channels become desensitized (daCosta et al., 2002; Hamouda et al., 2006b). Overall though, these studies show that nAChR receptor interaction with the surrounding lipids is a general phenomenon of lipid-protein interaction and whereas sterols clearly can associate with a channel protein, the specificity of these effects remained unclear. Significant effort, therefore, has been dedicated since late 1970s and until today to elucidate the nature of cholesterol interactions with nAChR.

### Annular and non-annular cholesterol binding sites

Two classes of lipid binding sites have been described: annular or boundary sites located on the transmembrane surface of a transmembrane protein, and non-annular sites located between transmembrane α-helices and occluded to phospholipids. ESR studies provided the first mechanistic insights into how cholesterol may interact with the channel proteins. First, it was revealed that the amount of lipids associated with the nAChR protein is significantly higher than would be required to form a single boundary shell around the protein. It was proposed that the immobilized lipids fill the space between the transmembrane helices of the protein and that this lipid layer may provide the medium of interaction between the subunits of the receptor (Marsh and Barrantes, 1978). This observation suggests that cholesterol may interact with the membrane proteins directly not only on the protein-bilayer interface but between the transmembrane subunits of the protein. Furthermore, analysis of the competition between cholesterol and phospholipids for the interaction with nAChR on the protein-bilayer interface using fluorescence quenching showed that cholesterol does not displace phospholipids from nAChR (Jones and McNamee, 1988). To account for this apparent discrepancy between the preferential association of nAChR with sterols demonstrated earlier and the inability of the sterols to displace phospholipids from the nAChR-lipid bilayer interface, Jones and McNamee (1988) proposed that cholesterol interacts with nAChR protein at *“non-annular” binding sites* that are not accessible to phospholipids. Clearly, this idea is fully consistent with the ESR analysis by Marsh and Barrantes (1978) showing that immobilized sterols may fill the space between the transmembrane helices.

Analysis of sterol ESR spectra in membranes containing digested segments of the protein provided further insights into the sterol-protein interactions of nAChR (Dreger et al., 1997). This study showed that enzymatic digestion of the extra-membrane domains of nAChR resulted in a significant decrease in the fraction of motionally restricted sterols consistent with the loss of sterol binding sites. In contrast, the fraction of the immobilized phospholipids was not affected by the digestion, providing additional evidence that cholesterol and phospholipids interact with nAChR through distinct binding sites. The requirement for the extra-membrane domains of the channel to support its interaction with cholesterol was interpreted as additional evidence for non-annular cholesterol binding sites (Dreger et al., 1997). A conclusion that cholesterol interacts with nAChR through non-annular sites was questioned, however, in view of the study of Addona et al. (1998) showing that tethering cholesterol molecule to the glycerol backbone of phosphatidylcholine does not prevent the functional impact of cholesterol on nAChR opening. Indeed, it is difficult to reconcile this observation with the idea of interstitial non-annular binding sites located within the channel protein. Addona et al. suggested, therefore, that functionally important cholesterol binding sites of nAChR must be close to the protein-lipid bilayer interface in what they called “peri-annular” locations. It is also possible, however, that the presence of cholesterol tethered to the glycerol backbone might alter the conformation of the protein resulting in a shift of the non-annular sites to a more peri-annular position.

Further evidence for direct interaction between cholesterol and nAChR came from studies using a photoactivatable cholesterol analog that cross-links with proteins upon UV illumination (Corbin et al., 1998; Hamouda et al., 2006a). These studies showed that the radio labeled photoactivatable cholesterol analog [^125^I]azido-cholesterol was incorporated into each of the nAChR subunits at equal molar ratios and that the amount of [^125^I]azido-cholesterol binding was found to be proportional to the surface of the protein-lipid interface leading the authors to conclude that cholesterol binds nAChR on the interface with the membrane lipids rather than at non-annular sites (Corbin et al., 1998). Further studies from the same group demonstrated that [^125^I]azido-cholesterol interacts with all transmembrane subunits (M1, M3, and M4) that contribute to the nAChR lipid protein interface, again emphasizing the correlation between cholesterol binding and the lipid-protein interface of nAChR and supporting the previous conclusion of the authors that cholesterol binds to annular rather than non-annular sites of nAChR (Hamouda et al., 2006a). A major constraint of these studies, however, was that, as pointed out by the authors, [^125^I]azido-cholesterol was added nAChR-containing membranes that already had a significant level of cholesterol. Therefore, if the non-annular cholesterol binding sites were already occupied by native cholesterol that was stably bound to these sites, they would have been inaccessible to the photoactivatable analog. Therefore, while these studies provide further evidence for direct interaction between cholesterol and nAChR, the discrimination between annular and non-annular sites was inconclusive. It was also pointed out in a later study (Brannigan et al., 2008) that incubation times used in these studies could be too short to allow labeled cholesterol to bind to internal non-annular sites. Furthermore, using molecular dynamics simulations, Brannigan et al. (2008) identified putative cholesterol-binding sites both at the protein-lipid interface of nAChR and sites that are deeply buried between the transmembrane subunits of the channels. Both sites were proposed to contribute to cholesterol functional effects on the nAChR channels. It is important to note, however, that the fact that cholesterol interacts directly with the protein does not necessarily mean that specific binding is required. Direct interaction of cholesterol with the channel protein might also alter channel function by the hydrophobic mismatch and increase in membrane deformation energy mechanisms.

## Specific vs. non-specific cholesterol-ion channels interactions: comparative sterol analysis

A powerful tool to discriminate between specific and non-specific effects of cholesterol on protein function is the substitution of endogenous cholesterol with sterols that have similar effects on the physical properties of the membrane. The rationale of this approach is that if cholesterol analogs have the same effects on the membrane structure and physical properties of the bilayer but differential effect on an ion channel function, it provides evidence for the specificity of the cholesterol effect. To achieve this goal, multiple studies used the substitution of cholesterol by one of its chiral analogs (enantiomers), a method based on the assumption that cholesterol enantiomers should have identical effects on the physical properties of the membrane but be strongly distinct in their ability to interact with the proteins. It is important to note, however, that while enantiomers have identical physical properties in an achiral environment, their effects on the physical properties of the lipid membranes depend on their interaction with other lipids and may not be identical because of the enantioselectivity of cholesterol-phsopholipid interactions [see Westover and Covey (2004) for review]. In spite of these constraints, two synthetic cholesterol analogs have been widely used to study the specificity of cholesterol-protein interactions: epicholesterol (3α-hydroxy-5-cholestene) that differs from natural cholesterol (3β-hydroxy-5-cholestene) in the rotational angle of the hydroxyl group at position 3 and *ent*-cholesterol that differs from natural cholesterol in the configuration of each of the eight stereocenters.

The rationale for using epicholesterol to substitute natural cholesterol in order to discriminate between specific and non-specific effects in cholesterol is based on the studies that showed that cholesterol and epicholesterol have similar effects on membrane fluidity (Gimpl et al., 1997) and on the formation of structured lipid domains (Xu and London, 2000). However, while cholesterol has a significant condensing effect on the areas of other lipids, epicholesterol has a weaker or no effect (Demel et al., 1972a,b). Cholesterol and epicholesterol also have different orientation (tilt angle) within the lipid bilayer and while cholesterol is tilted in parallel to the phospholipids, epicholesterol resides more perpendicular to the surface of the bilayer (Murari et al., 1986). These studies suggest that the two sterols might have differential effects on the lipid packing of the membrane. In many studies, therefore, as described below, cholesterol was substituted not only by epicholesterol but by an array of different sterols to determine the correlation between the impact of the sterols on the physical properties of the membrane and on the activity of a specific protein. Clearly, the benefit of the latter approach is that it allows to directly test for correlation between the two effects in a given system. More recently, there is growing interest in using *ent*-cholesterol that appears to be the closest to natural cholesterol in terms of its impact on the physical properties of the lipid bilayer including membrane thickness, a parameter that might be more critical for protein function than membrane fluidity and compression behavior (Mickus et al., 1992; Rychnovski and Mickus, 1992; Westover and Covey, 2004). Here, we describe how different sterol substitutions have been used to determine the specificity of cholesterol interactions with various ion channels. All the effects are summarized in Table 1.

**Table 1:** **Sterol specificity of cholesterol effects on different types of ion channels**.

**Channel**	**Assay**	**Substitutions**			**Conclusion**
		**Chol/Epichol**	**Chol*Ent*-chol**	**Other sterols/correlation with physical properties of the bilayer**	
nAChR	Incorporation into films	No selectivity	–	No correlation	Specific (Popot et al., 1978)
	Agonist-induced activity	No selectivity	No selectivity	No correlation	Specific (Addona et al., 2003)
GABA_*A*_	Agonist-induced activity	Partial specificity	–	–	Specific (Sooksawate and Simmonds, 2001)
VRAC	Swelling-induced activity	No selectivity	–	Correlation	Nonspecific (Romanenko et al., 2004b)
Kir channels					
Kir2.1	Whole-cell current	Stereospecificity	–	–	Specific (Romanenko et al., 2002)
	Activity in liposomes	–	Stereospecificity	–	Specific (D'Avanzo et al., 2010)
KirBac1.1	Activity in liposomes	Stereospecificity	–	No correlation	Specific (Singh et al., 2009)
	Binding	No selectivity	–	–	Specific (Singh et al., 2011)
BK channels	Activity in bilayers	Stereospecificity	Stereospecificity	No correlation	Specific (Bukiya et al., 2011)
TRPV1	Capsaicin-induced current	Stereospecificity	–	–	Specific (Picazo-Juarez et al., 2011)

### Nicotinic acetylcholine receptor (nAChR)

An early study of Popot et al. (1978) used this approach to analyze the ability of nAChR to be incorporated into monolayer lipid films that contained different sterol analogs, including cholesterol, ergosterol, epicholesterol, androstenol, stigmasterol, and coprostenol. Using this approach, Popot et al. showed that the ability of nAChR to be incorporated into the monolayers containing cholesterol was significantly higher than that for monolayers containing ergosterol while both sterols were shown earlier to have the same effects on membrane permeability to small molecules (Demel et al., 1972a). This observation was interpreted as evidence for direct interaction of cholesterol with nAChR (Popot et al., 1978). No significant difference, however, was observed between nAChR incorporation into monolayers containing cholesterol and epicholesterol. This early study suggested, therefore, that both cholesterol and epicholesterol may interact with the nAChR protein.

These findings were confirmed by a more recent study of Addona et al. (2003) who showed that similarly to cholesterol, epicholesterol can support the functional activity of nAChR. Furthermore, the same results were observed when cholesterol was substituted with *ent*-cholesterol. Based on the original premise of this approach, these observations could have been interpreted as evidence that nAChR is regulated by the physical properties of the membrane rather than by direct interactions between the sterols and the protein. However, Addona et al. also found that nAChR functional activity can also be supported by coprastanol, a sterol that lacks the planar structure that is critical for the typical effects of cholesterol on lipid bilayer. Taken together with the earlier studies that showed immobilization of cholesterol molecules by nAChR protein described above, the conclusion was that cholesterol binds directly to the nAChR but that the binding site has very lax structural requirements and the binding is not stereo-selective (Addona et al., 2003). Interestingly, new mechanistic insights into the role of cholesterol in the regulation of nAChR were obtained by a recent study of daCosta et al. (2013) showing that cholesterol regulates the channels by two distinct mechanisms: stabilization of the channels in a resting state that depends on specific lipid-protein interactions and facilitation of the transitions between uncoupled and coupled states that depends on the hydrophobic thickness of the membrane. A detailed analysis of cholesterol binding motifs that are responsible for cholesterol interaction with the nAChR is provided in later sections of this review.

### GABA_A_ receptor

Similarly to nAChR, cholesterol was shown to be required to support agonist-induced opening of the GABA_A_ receptor (Sooksawate and Simmonds, 2001), a member of a superfamily of ligand-gated ion channels that forms a chloride channel. In this case, however, the effect was stereospecific because repletion of cholesterol-depleted cells with epicholesterol failed to support GABA_A_ function. In addition, the authors showed that not only cholesterol depletion but also cholesterol enrichment has a detrimental effect on GABA_A_function but the latter appeared to be non-specific. The authors concluded that GABA_A_ receptor is regulated by cholesterol by both specific and non-specific mechanisms. It was also suggested that since nAChR and GABA_A_ belong to a superfamily of *Cys*-loop receptors, the same mechanisms may apply to cholesterol regulation of the two proteins (Brannigan et al., 2008). Clearly, more studies are needed to test this prediction.

### Volume-regulated anion channels (VRAC)

In contrast to nAChR and GABA_A_ receptors, VRAC channels were shown to be suppressed by an increase in membrane cholesterol and enhanced by cholesterol depletion (Levitan et al., 2000; Klausen et al., 2006). The channels are activated in response to an osmotic gradient that causes cell swelling and the effect of cholesterol depends on the intensity of the stimulus: when cells are challenged with a mild osmotic gradient, cholesterol strongly suppresses the currents but when cells are exposed to a high gradient, no more inhibition is observed, suggesting that strong stimulus overcomes cholesterol-induced inhibition (Levitan et al., 2000). Furthermore, we showed that substitution of cholesterol with epicholesterol has no impact on VRAC activity indicating that cholesterol effect on VRAC is not stereoselective (Romanenko et al., 2004b). Substituting cholesterol with coprastanol, however, had a significant effect which is consistent with the sensitivity of the channels to lipid packing.

### Inwardly-rectifying K^+^ channels (Kir)

**Figure 1:**
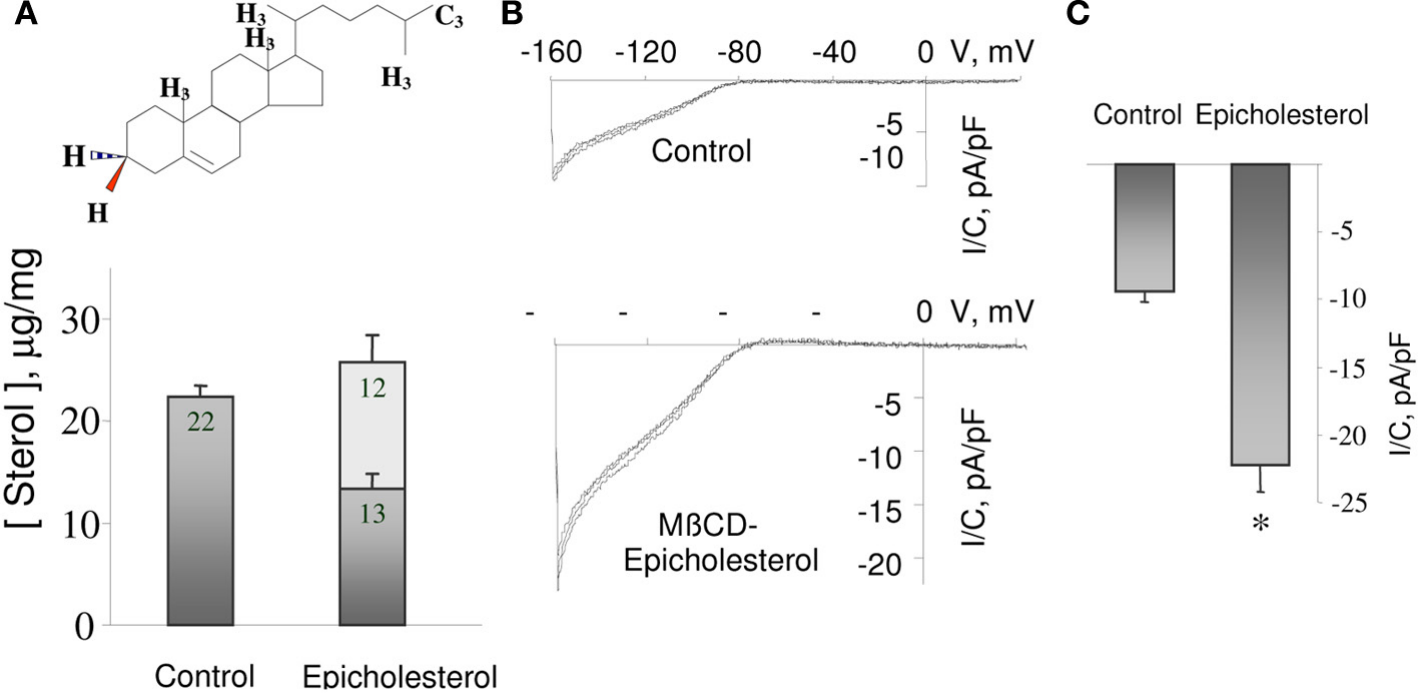
**Substitution of cholesterol by epicholesterol increases Kir current density. (A)** Substitution of endogenous cholesterol by epicholesterol using MβCD. Dark bars represent the level of cholesterol, and the lighter portion of the bar, epicholesterol level. **(B)** Typical current traces recorded from a cell exposed to Mβ CD-epicholesterol and from a control cell. Both cells were maintained in 6 mM extracellular K^+^. **(C)** Peak current densities of MβCD-epicholesterol treated cells (*n* = 32) and in control cells (*n* = 31) recorded in 6 mM extracellular K^+^. All values are means ± SE ^*^P < 0.05 vs. control. From Romanenko et al. (2002).

Our studies also showed that Kir channels are strongly suppressed by an increase in membrane cholesterol and enhanced by cholesterol depletion (Romanenko et al., 2002, 2004a). This effect was observed in aortic endothelial cells *in vitro* and *in vivo* (Romanenko et al., 2002; Fang et al., 2006; Mohler et al., 2007), cardiomyocytes (Deng et al., 2012), macrophages (not published) and in several expression systems (Romanenko et al., 2004a; Rosenhouse-Dantsker et al., 2010). Unexpectedly, substituting ~50% of endogenous cholesterol with epicholesterol resulted in a significant increase in endothelial Kir currents beyond the effect of cholesterol depletion (Figure 1; Romanenko et al., 2002). The reason that it was a surprising finding is that it did not fit neither with the scenario that cholesterol regulates the channels by altering the physical properties of the membrane, nor with the scenario that it binds to the channel protein in a stereo-selective way. Indeed, if cholesterol regulates Kir by altering the physical properties of the membrane than substituting it with a sterol that has similar properties should have no effect. On the other hand, if cholesterol regulates Kir channels by stereo-specific binding, then epicholesterol is expected to be “invisible” for the channels and cholesterol-epicholesterol substitution should have a “depletion” effect. Instead, we proposed that, similarly to nAChR, cholesterol regulates Kir channels by direct interaction with the channel protein and that this interaction is not stereospecific. However, in contrast to nAChR, we proposed that while the binding of the sterols to Kir channels is not stereospecific, the functional effect of the binding is. That means that while epicholesterol might be able to bind to Kir channels, it does not have an inhibitory effect. Furthermore, in this case, cholesterol and epicholesterol might compete for the same binding sites which would explain epicholesterol-induced increase in Kir activity.

**Figure 2:**
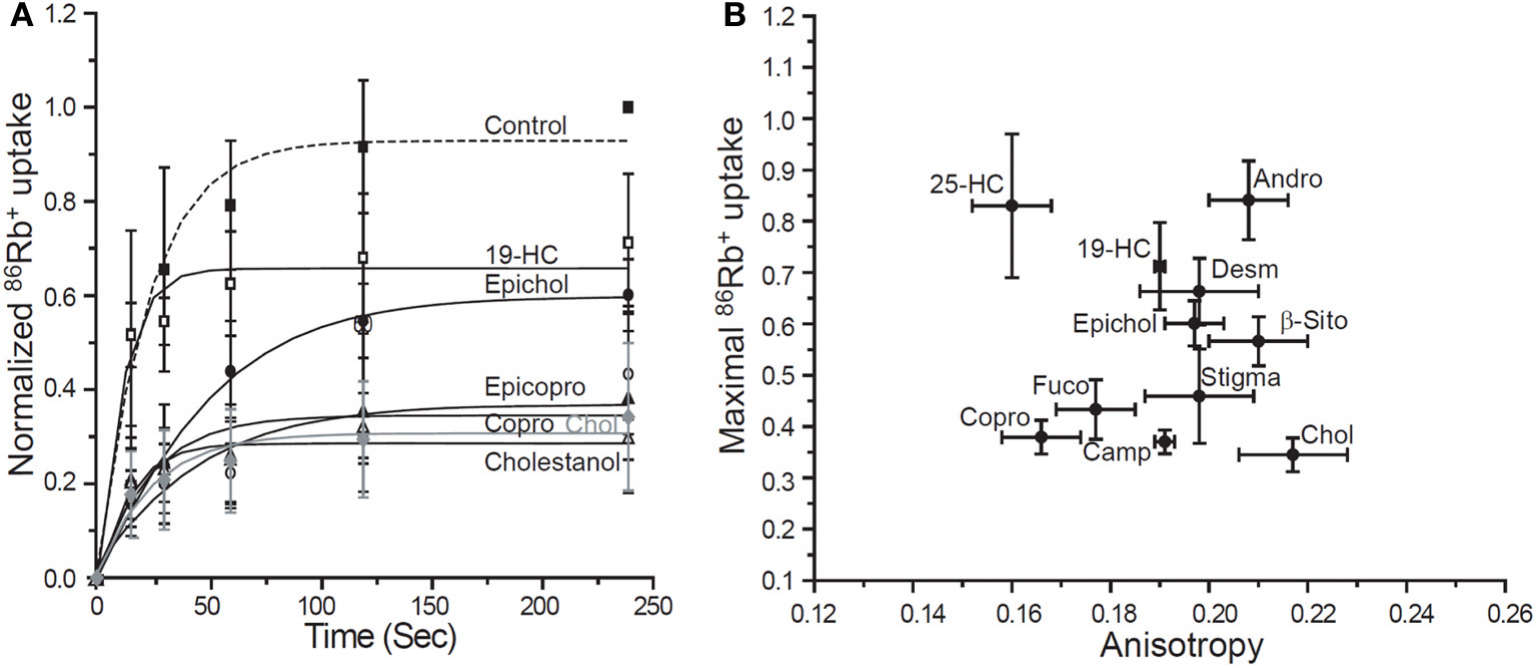
**Differential effects of sterol on KirBac1.1-mediated ^86^Rb^+^ uptake. (A)** Time-courses of ^86^Rb^+^ uptake in liposomes containing different sterols. All experiments included control liposomes containing no sterol and liposomes containing 50 μg cholesterol/mg PL as a positive control. **(B)** KirBac1.1 activity vs. membrane anisotropy. The normalized maximal uptake of ^86^Rb^+^ isplotted vs. the anisotropy (r) following incorporation of respective sterols. The correlation coefficient between the ^86^Rb^+^ uptake and the anisotropy value is *R* = −0.08276, *p* > 0.05 (a meaningful correlation would require |*R*| > 0.602) (Abbreviations: 25-HC, 25-Hydroxycholesterol; Desm, Desmosterol; β-Sito, β-Sitosterol; Camp, Campesterol; Fuco, Fucosterol; Chol, Cholesterol; Copro, Coprosterol; 19-HC, 19-Hydroxycholesterol; Epicopro, Epicoprosterol; Epichol, Epicholesterol; Andro, 5-Androsten 3β-17 β-diol; Ergo, Ergosterol; Stigma, Stigmastanol). From Singh et al. (2009).

Sensitivity of the channels to the chiral structure of cholesterol in the complex environment of the plasma membrane, however, may not necessarily mean that cholesterol interacts directly with the channels. Obviously, cholesterol may affect channels function by interacting with other proteins, which in turn may regulate the activity of the channels. To address this issue, we tested cholesterol sensitivity of purified bacterial K^+^ channels, KirBac1.1 incorporated into liposomes that have no other protein components (Singh et al., 2009). KirBac channels have been used in multiple studies as structural models of mammalian Kir channels because of their high sequence homology to mammalian Kirs (e.g., 52% homology between KirBac1.1 and Kir2.1) (e.g., Bichet et al., 2003; Kuo et al., 2003). Our study showed that no intermediates are required for the effect of cholesterol on KirBac1.1 channels (Singh et al., 2009). Furthermore, structural analysis of KirBac1.1 sensitivity to multiple sterols showed that there is no correlation between the effects of the sterols on KirBac1.1 and on membrane fluidity indicating that membrane fluidity cannot account for the effects of the sterols on KirBac1.1 activity (Figure 2; Singh et al., 2009). Consistent with these observations, D'Avanzo et al. (2011) showed that cholesterol also suppresses the function of purified mammalian Kir2.1 channels and that this effect is lost when cholesterol is substituted with *ent*-cholesterol. Importantly, the latter observation indicates that *ent*-cholesterol does not support the functional effect of cholesterol on Kir channel but it is fully compatible with the idea that sterol analogs of cholesterol may bind to the channel protein but fail to exert a functional effect. Our further studies demonstrated that cholesterol indeed binds to purified KirBac1.1 channels (Singh et al., 2011) and most recently we provided a first comprehensive analysis of putative cholesterol binding sites of Kir2.1 channels (Rosenhouse-Dantsker et al., 2013). These studies are described in later sections of this review.

### Large-conductance Ca^2+^-sensitive voltage-gated K^+^ channels (BK)

Several studies demonstrated that elevation of membrane cholesterol inhibits BK activity both in native cell membranes (Bolotina et al., 1989) and in reconstituted bilayers (Crowley et al., 2003; Bukiya et al., 2008, 2011). Initially, it was proposed that cholesterol-induced inhibition of BK channels should be attributed to a decrease in membrane fluidity (Bolotina et al., 1989) or increase in bilayer lateral stress (Chang et al., 1995). More recent studies of Dopico and colleagues, however, demonstrated that this is not the case. Specifically, Bukiya et al. (2011) showed that substituting cholesterol with epicholesterol resulted in a complete loss of sterol-induced inhibition of BK channel activity. Furthermore, substitution of cholesterol with *ent-cholesterol* had the same effect: in contrast to cholesterol, incorporation of either epicholesterol or *ent*-cholesterol into the bilayer had no effect on the activity of BK channels. Remarkably, substitution of cholesterol with coprastanol or with cholestanol had the same effect on BK function as cholesterol. This observation is very significant because, as described above, coprastanol lacks the planar structure that is important for the condensation effect of the sterol on lipid packing. Indeed, coprastanol was shown to disrupt lipid packing and have “anti-condensation effect” (Xu and London, 2000; Bukiya et al., 2011). Thus, the ability of coprastanol to have the same effect on channel function as that of cholesterol indicates that this effect cannot be attributed to changes in lipid packing. Interestingly, the ability of coprastanol to inhibit BK channels is abolished when it is substituted with epicoprastanol showing that this effect is also stereospecific. Taken together, these observations led to the conclusion that cholesterol regulates BK channels by specific sterol-protein interactions and not by changing the physical properties of the membrane. Furthermore, similarly to the studies described above for nAChR and Kir channels, the structural requirements for cholesterol interaction with the BK channels are expected to be lax.

### Transient receptor potential vanilloid 1 channels (TRPV1)

Cholesterol was also shown to have a significant impact of different types of Transient Receptor Potential (TRP) channels, including TRPV1. Earlier studies demonstrated that cholesterol depletion results in strong suppression of the capsaicin-induced whole cell currents and it was suggested that TRPV1 channels need a cholesterol-rich environment of a lipid raft for their function (Liu et al., 2006; Szoke et al., 2010). This conclusion though was challenged in a later study by Rosenbaum and colleagues (Picazo-Juarez et al., 2011) who used excised patches to test the effect of cholesterol on TRPV1 channels. This study showed that cholesterol had no effect on TRPV1 channels and that cholesterol enrichment had a strong inhibitory effect. The discrepancy between this and earlier studies was explained by a possible effect of cholesterol on TRPV1 trafficking to the membrane which could have masked the effect of cholesterol on the individual channels. In terms of the specificity of cholesterol-induced inhibition of TRPV1 channels, Picazo-Juarez et al. showed that substitution of cholesterol by epicholesterol abrogated the inhibitory effect indicating that this effect is stereoselective.

*In summary*, most types of ion channels analyzed today for the specificity of their interaction with cholesterol appear to be regulated by specific sterol-protein interactions. It is important to note, however, that the specificity may not manifest itself in the stereoselectivity of the responses, as it was shown for the nicotinic AChR (Addona et al., 2003). Multiple criteria, therefore, should be used to determine whether this is the case. Furthermore, given a possible lack of stereospecificity of cholesterol interaction with ion channels, it is imperative to develop further assay that test cholesterol-ion channel binding directly.

## Direct binding of cholesterol to ion channels

### Evidence for direct binding of cholesterol to KirBac channels

**Figure 3:**
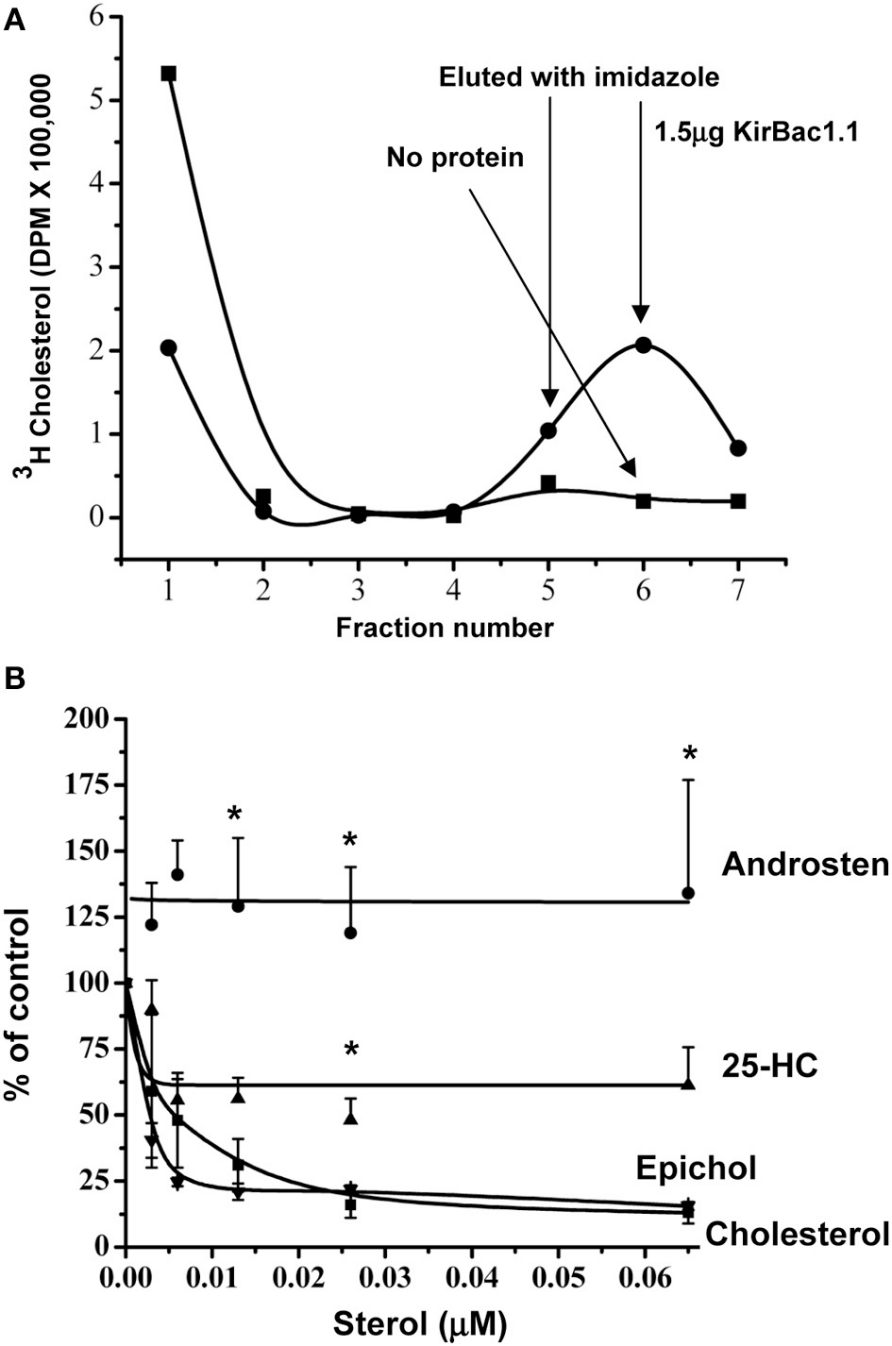
**Specific cholesterol binding to KirBac1.1. (A)** Typical elution profiles of [^3^H]-cholesterol with and without 1.5 μg His_6_-KirBac1.1 protein from Ni-NTA-agarose affinity column. The unbound cholesterol is eluted in fractions 1–4 and cholesterol bound to the KirBac1.1 protein is eluted in fractions 5–7 after the addition of imidazole-HCl. **(B)** Competition between ^3^H-cholesterol and unlabeled cholesterol, epicholesterol, 25-Hydroxycholesterol and, 5-Androsten 3β-17 β-diol. From Singh et al. (2011). ^*^p < 0.05.

Direct evidence for cholesterol binding has been demonstrated so far only for a small number of proteins (Gimpl, 2010). Traditionally, binding studies are performed by incubating a protein with a radiolabeled ligand and then separating the bound and free ligand by different centrifugation or filtration techniques. This approach, however, is significantly more challenging for water insoluble ligands, such as cholesterol, because organic solvents might interfere with specific sterol-protein interactions of membrane proteins (Radhakrishnan et al., 2004; Gimpl, 2010). Thus, the main constraint in establishing a cholesterol binding assay is solubilizing cholesterol without disrupting the binding ability of the protein. Earlier studies have shown that this problem can be successfully resolved by solubilizing cholesterol in detergent micelles of either Fos-Choline 13 or Nonidet P-40 and using His-tagged proteins that can be separated on nickel agarose columns (Radhakrishnan et al., 2004; Infante et al., 2008a,b). Using this approach, saturation and kinetic data for cholesterol binding was obtained for two major cholesterol binding proteins, SCAP1 and NPC1 (Radhakrishnan et al., 2004; Infante et al., 2008a,b). We used the same approach, therefore, to analyze cholesterol binding to purified KirBac channels. In this case, we used another detergent, 3-[(3-Cholamidopropyl) dimethyl-ammonio]-propanesulfonate hydrate (CHAPS) for cholesterol solubilization, which we have already shown earlier not to interfere with KirBac1.1 function and its sensitivity to cholesterol (Singh et al., 2009). Indeed, we have found that cholesterol binds to KirBac1.1 protein in a reproducible way, as is demonstrated by a typical [^3^H]cholesterol elution profile (Figure 3A). As expected, the profile consists of two clear peaks that correspond to the unbound (fractions 1–4) and *bound cholesterol* (fractions 5–7).

### Saturability

We also found that the binding is clearly saturable but the saturation occurred at relatively high cholesterol concentration with the K_*D*_ estimated to be ~400 μM (Singh et al., 2011). This is significantly higher than the K_D_ values estimated for SCAP and NPC proteins that range between 30 nM and ~2 μM (Gimpl, 2010). It is important to note, however, that the environment of the plasma membrane where Kir channels are functional is also significantly richer in cholesterol than the intracellular membranes that harbor SCAP and NPC proteins. It would make sense, therefore, for Kir channels to have lower affinity to cholesterol than the intracellular proteins. It is important to note, however, that KirBac is a bacterial protein that is not exposed to cholesterol in its natural environment. The fact that KirBac still binds cholesterol and is functionally regulated by changes in cholesterol levels provides further support for the structural-functional homology between bacterial and mammalian Kir channels. Moreover, the high K_D_ value suggests that most likely cholesterol interacts with the channel protein by weak hydrophobic association. It is also interesting to note that bacterial channels are not exposed to cholesterol in nature. In terms of the functional significance, therefore, one possibility is that the channels may interact with other sterols that are present in bacteria but this has not been studied. Another possibility is that the general structure of multiple membrane proteins including bacterial ion channels has hydrophobic surfaces in between their transmembrane helices that are capable to bind cholesterol molecule. In the presence of cholesterol, such surfaces may develop into regulatory sites.

### Competition with other sterols: no difference between cholesterol and epicholesterol

To examine the specificity of sterol binding to KirBac channels, we tested three sterols: epicholesterol, 5-Androsten 3β-17β-diol and 25-hydroxycholesterol. Our previous studies showed that epicholesterol is significantly less efficient than cholesterol in suppressing KirBac activity and androsten and 25-hydroxycholesterol do not affect the channels at all (Singh et al., 2009). These experiments showed that while epicholesterol binding to KirBac1.1 was similar to that of cholesterol, 25-hydroxycholesterol binding was significantly weaker and 5-Androsten 3β-17β-diol did not compete with cholesterol at all (Figure 3B). Thus, cholesterol binding to the KirBac protein is clearly specific. The most significant and novel result of this series of experiments was that epicholesterol was found to bind to the channel protein as efficiently as cholesterol. The novelty of this observations is *not* in the fact that epicholesterol was found to interact with a protein. Indeed, as described above, both chiral analogs of cholesterol, epicholesterol, and *ent*-cholesterol were found to interact with nAChR (Addona et al., 2003). Epicholesterol was also found to bind to a sterol-sensing domain of SCAP (Radhakrishnan et al., 2004). The major difference, however, is that in nAChR epicholesterol is also capable of substituting for cholesterol in terms of its functional effect on the channel whereas in Kir channels, this is not the case.

*In summary*, these studies provide proof of principle that specific saturable binding of cholesterol to an ion channel can be detected using the appropriate biochemical techniques and confirm that Kir channels can be regulated by specific sterol-protein interactions. These studies also provide novel insights into the differential effects of sterols on channel function by raising an important question about the interpretation of comparative analysis of sterol analogs on ion channel function. In contrast to a common interpretation that differential effects of cholesterol enantiomers on channel function indicate a lack of analog binding, our observations suggest that chiral analogs may bind to the channel protein but have no inhibitory effect. The latter interpretation is also supported by our earlier studies showing that cholesterol and epicholesterol have opposite effects on Kir currents suggesting that the two analogs compete for a binding site (Romanenko et al., 2002).

## Structural features of cholesterol interaction with ion channels

As described above, earlier studies provided evidence for non-annular cholesterol binding regions in nAChR (Jones and McNamee, 1988) as described earlier in this review, and in Ca^2+^-ATPase of sarcoplasmic reticulum (Simmonds et al., 1982). More recently, X-ray crystallography revealed cholesterol molecules bound between the transmembrane helices of several G-protein coupled receptors (GPCRs) (Yeagle, 2013). For example, several cholesterol molecules were found between four transmembrane helices (I, II, III, and IV) of the β-Adrenergic GPCR (Cherezov et al., 2007; Hanson et al., 2008) as well as a cholesterol molecule wedged within a groove formed by three transmembrane helices (Ia, V, and VII) of the dopamine receptor (Penmatsa et al., 2013). A cholesterol binding site was also identified in the 5-HT_2B_ERG receptor (Wacker et al., 2013) and three cholesterol binding sites were found in the crystal structure of human A2A adenosine receptor (Liu et al., 2012). Additional examples include also two cholesterol molecules that were observed in the proton pumping Rhodopsin, ARII, between the transmembrane helices of two adjacent proteins (I and VII in one protein and I in the adjacent protein) (Wada et al., 2011), and one cholesterol molecule that was seen in the structure of the μ-opiod receptor in between the transmembrane helices of two receptors (Manglik et al., 2012). Most recently, our studies identified two putative non-annular cholesterol binding regions in Kir2.1 channels (Rosenhouse-Dantsker et al., 2013). The goal of this part of the study is to describe the structural features of cholesterol binding motifs and putative binding regions in ion channels.

### Cholesterol binding motifs: CRAC, CARC, and CCM

Three motifs have been previously associated with cholesterol binding to transmembrane proteins. The most well-known motif is the cholesterol recognition amino acid consensus (CRAC) motif, which is -L/V-(X)(1–5)-Y-(X)(1–5)-R/K- where (X)(1–5) represents between one and five residues of any amino acid (Li and Papadopoulos, 1998; Epand, 2006). Accordingly, cholesterol binding requires a bulky hydrophobic residue (leucine or valine), the aromatic residue tyrosine, and a positively charged residue (arginine or lysine). Recently, an inverted CRAC motif, the CARC motif, has been shown to be more consistent in predicting cholesterol recognition motifs in integral membrane proteins (Fantini and Barrantes, 2013). As its name hints, the order of the required residues for cholesterol binding is inverted in the CARC motif compared with their order in the CRAC motif. Additionally, in this motif the tyrosine can be replaced by a different aromatic residue, a phenylalanine. In summary, the CARC motif is R/K-(X)(1–5)-Y/F-(X)(1–5)-L/V. The third established cholesterol binding motif is the cholesterol consensus motif (CCM) (Hanson et al., 2008). Unlike the CRAC and CARC motifs that include residues from one continuous segment of the protein, the CCM includes residues on adjacent helices: (W/Y)-(I/V/L)-(K/R) on one helix, and (F/Y/R) on the second helix. Notably, the types of residues included in the CCM are similar to those in the CRAC and CARC motifs. In recent years, both the CRAC motif and its inverted version, the CARC motif, have been found in several ion channels. These include the transient receptor potential TRPV1 channels (Picazo-Juarez et al., 2011), the large conductance Ca^2+^ and voltage-gated K^+^ (BK) channels (Singh et al., 2012), the nicotinic acetylcholine receptor (nAChR) (Fantini and Barrantes, 2013), and the inwardly rectifying potassium channel Kir2.1 (Rosenhouse-Dantsker et al., 2013).

#### CRAC motifs

*CRAC motifs* were found in BK, nAChR, and Kir2.1 channels. In BK, seven CRAC motifs were found in the cytosolic domain (Singh et al., 2012) with the most pronounced effect on the sensitivity of the channel to cholesterol found in the membrane-adjacent CRAC4 motif, V444—Y450—K453. In addition, cumulative truncations or Y-to-F substitutions in CRAC5 to CRAC10 progressively decreased the sensitivity of the channel to cholesterol, demonstrating the role of multiple CRACs in the sensitivity of BK channels to cholesterol. A CRAC motif was also found in AChR subunits in the region immediately adjacent to TM1 and stretching out into the extracellular domain of the AChR but in view of its location outside the membrane bilayer, it was suggested that this CRAC motif would not be energetically favorable for cholesterol binding (Fantini and Barrantes, 2013). Similarly, a CRAC motif was also found in Kir2.1 outside the transmembrane domain, in a cytosolic segment that is highly energetically unfavorable for cholesterol binding (Rosenhouse-Dantsker et al., 2013).

#### CARC motifs

*CARC motifs*, on the other hand, were identified in the transmembrane domain of TRPV1 (Picazo-Juarez et al., 2011), AChR (Fantini and Barrantes, 2013), and Kir2.1 (Rosenhouse-Dantsker et al., 2013) providing more favorable putative cholesterol binding sites. In TRPV1, the R-(X)(2)-F-(X)(2)-L CARC sequence R579—F582—L585 is located in the S5 transmembrane helix of the channel. Mutations of the three characteristic CARC motif residues affected the sensitivity of TRPV1 to cholesterol (Picazo-Juarez et al., 2011). In AChR, three cholesterol molecules could be docked on the TM1, TM3, and TM4 transmembrane helices of each AChR subunit (Fantini and Barrantes, 2013). All three sites corresponded to CARC motifs, rendering a total of 15 possible cholesterol binding sites per AChR molecule. In Kir2.1, there are several CARC motifs in both the cytosolic and transmembrane domain. Aside from the CARC motifs that are located in the cytosolic domain and are unfavorable for cholesterol binding, Kir2.1 has two CARC motifs at the interface between the transmembrane and cytosolic domains (Rosenhouse-Dantsker et al., 2013). These include (1) R67—F73—V77 and (2) R82—F88—L90. In the first CARC motif, the V77I mutation resulted in loss of cholesterol sensitivity. However, the roles of the other two residues (R67 and F73) could not be tested because their mutations resulted in non-functional channels. In contrast, mutations of all three residues of the second CARC motif, did not affect the sensitivity of the channel to cholesterol suggesting that it does not describe a cholesterol binding site in these channels. The existence of a CRAC/CARC motif does not necessarily imply, however, that cholesterol would bind to the protein at the corresponding region. Furthermore, it is also possible that the three cholesterol binding motifs described above do not account for all the possible cholesterol-protein interactions, and that cholesterol may interact with ion channels through previously unidentified interaction motifs.

### Novel cholesterol binding regions in kir2.1

#### A lack of putative cholesterol binding sites at the annular sites of the transmembrane domain and in the cytosolic domain of Kir2.1 channels

We have shown earlier that mutations of residues located at the lipid-protein interface of the transmembrane domain do not affect cholesterol sensitivity of Kir2.1 suggesting that cholesterol does not bind to annular sites of Kir2.1 (Epshtein et al., 2009). We have also identified a series of residues in the cytosolic domain of Kir channels that significantly reduced or abrogated the sensitivity of Kir2.1 to cholesterol but based on the docking analysis, our conclusion was that these residues do not constitute a cholesterol binding site (Epshtein et al., 2009; Rosenhouse-Dantsker et al., 2011; Rosenhouse-Dantsker and Levitan, 2012). Specifically, we first identified several residues in the CD loop of the cytosolic domain of the channel (N216, K219, and L222) (Epshtein et al., 2009) and then showed that these residues are a part of a group of residues that form a belt structure surrounding the cytosolic pore of the channel close to its interface with the membrane (Rosenhouse-Dantsker et al., 2011). Yet, there was no correlation between the location of the cholesterol sensitivity belt and any potential cholesterol binding sites obtained from docking analysis. Rather, all potential binding sites were located either above or below the plane of the cholesterol sensitivity belt (Rosenhouse-Dantsker et al., 2011) Our conclusion was therefore, that cholesterol sensitivity belt does not constitute a cholesterol binding site but regulates cholesterol sensitivity of the channels by affecting channel gating. Thus, no putative cholesterol binding sites were found neither at the annular sites of the transmembrane domain nor in the cytosolic domain of Kir2.1 channels.

#### Identification of novel non-annular cholesterol binding regions in Kir2.1 channels

**Figure 4:**
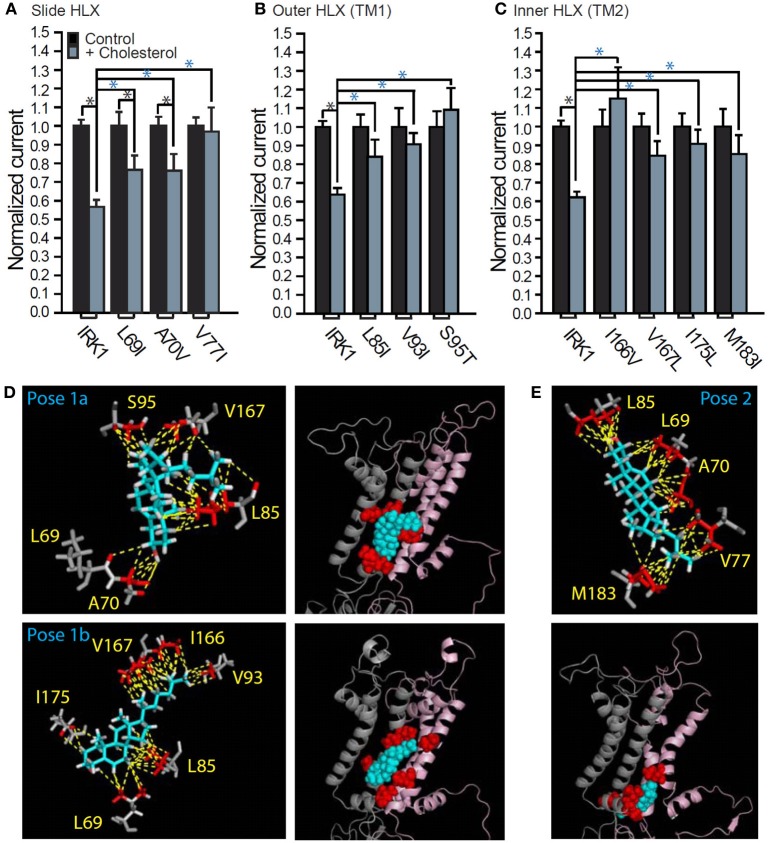
**Cholesterol recognition residues in the two putative transmembrane binding regions in Kir2.1.** Whole-cell basal currents recorded in *Xenopus* oocytes at −80 mV showing the effect of cholesterol enrichment on Kir2.1 and **(A)** L69I, A70V, and V77I of the slide helix, **(B)** L85I, V93I, and S95T of the outer helix, and **(C)** I166V, V167L, I175L, and M183I of the inner helix (*n* = 9–90). Significant difference is indicated by an asterisk (^*^p ≤ 0.05). A black asterisk indicates significant difference between whole-cell currents obtained for same construct following cholesterol enrichment and in the absence of treatment (control). A blue asterisk indicates significant difference between the effect of cholesterol enrichment on a mutant and the WT Kir2.1 channel. **(D,E)** Stick and ball presentations of the cholesterol recognition residues that surround the cholesterol molecule in each of the two putative binding regions after 50 ns of all-atom full-membrane molecular dynamics simulations. Two representative poses in region 1 are shown in **(D)** and one representative pose in region 2 is shown in **(E)**. The relative position of each binding regions in the TM domain of the channel is shown in the ball presentation that includes the two adjacent subunits of the channel that interact with the cholesterol molecule in a cartoon presentation. From Rosenhouse-Dantsker et al. (2013).

**Figure 5:**
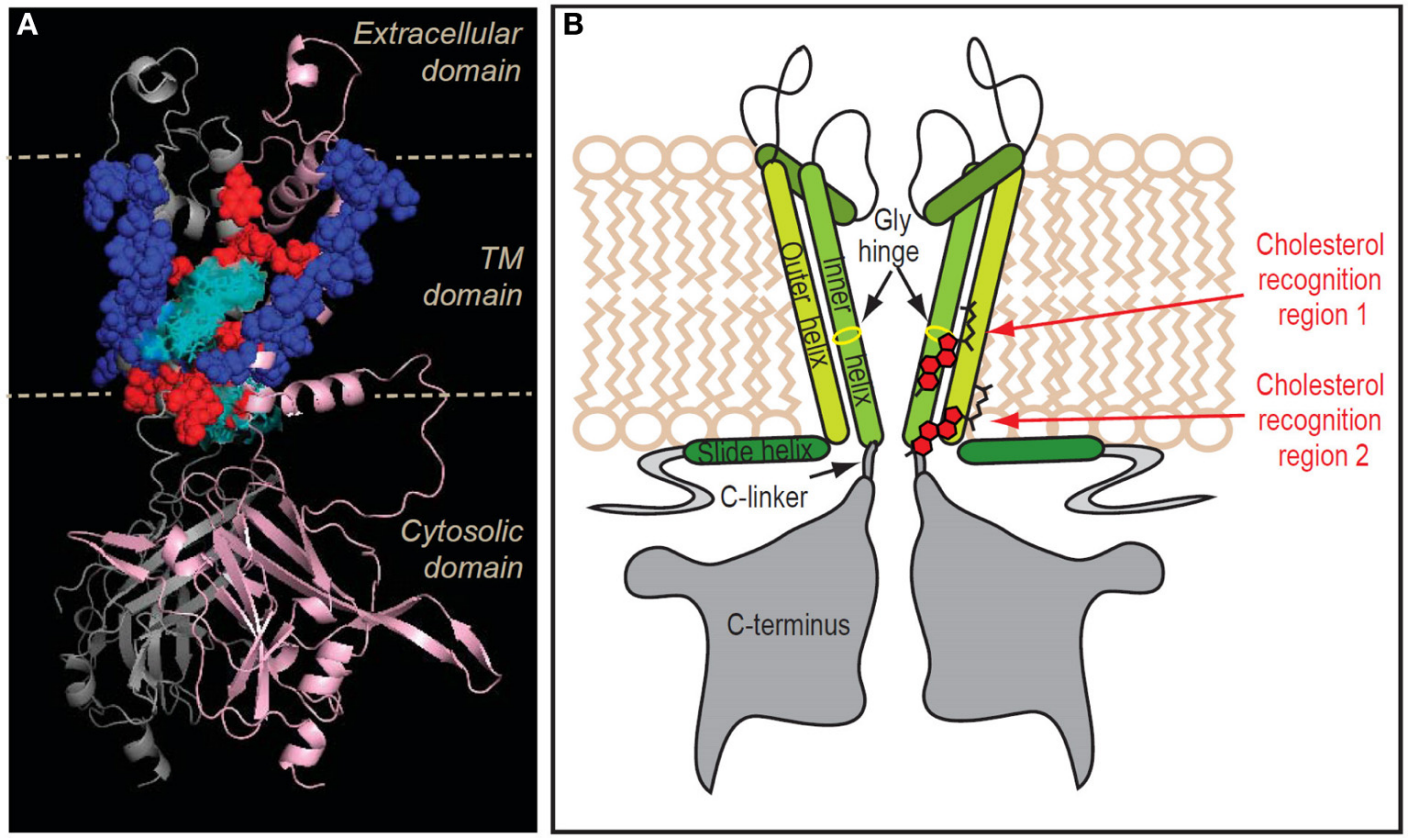
**Location of the putative transmembrane cholesterol binding regions in Kir2.1. (A)** Ribbon presentation of two adjacent subunits of Kir2.1 (pink and gray) showing the TM residues whose mutation affects the sensitivity of the channel to cholesterol (in red balls) relative to the location of the five cholesterol sites (in cyan sticks and surface presentation). Also shown are the continuous chains of residues that border the cholesterol binding groove in the channel (in blue balls). **(B)** Schematic model illustrating the location of the two cholesterol binding regions along with labeling of the channel regions. Note that for clarity purposes, the model shows the cholesterol molecules next to one of the two adjacent channel subunits with which they are predicted to interact. From Rosenhouse-Dantsker et al. (2013).

To identify possible non-annular cholesterol-binding sites in Kir2.1, we used a combination of docking studies, all-atom molecular dynamics simulations and site-directed mutagenesis, an approach that led to the identification of two novel putative cholesterol binding regions (Rosenhouse-Dantsker et al., 2013). One binding region is located in the center of the transmembrane domain of Kir2.1 (region 1), and the second—at the interface between the transmembrane and cytosolic domains of the channel (region 2) (Figures 4, 5). Within region 1 we identified 8 residues whose mutation abrogated or significantly decreased the sensitivity of the channel to cholesterol (Figures 4A–D). These residues are primarily bulky hydrophobic (I, L, or V), but also include a polar uncharged serine residue. Mutations of several aromatic and positively charged residues located in this region resulted in a non-functional channel or did not have any effect on the sensitivity of the channel to cholesterol. Their role in cholesterol binding, however, cannot be excluded because mild mutations may not sufficiently affect the interaction of the channel with the cholesterol molecule. Notably, the 8 residues in region 1 whose mutation affected the sensitivity of the channel to cholesterol were distributed among the α-helices of two adjacent subunits of the channel. Accordingly, the cholesterol molecule would bind in between these α-helices. Within region 2, we identified 5 hydrophobic residues (A, L, V, and M) whose mutation abrogated or significantly decreased the sensitivity of the channel to cholesterol (Figures 4A–C,E). Similarly to the cholesterol binding pocket in region 1, these 5 residues are located in two adjacent subunits of the channel indicating that also in this region, cholesterol would bind in between the α-helices. Furthermore, based on all-atom full-membrane 50 ns molecular dynamics simulations, cholesterol molecule exhibited significant flexibility, continuously exploring a considerable conformational space within each binding region, and interacting with different sets of residues in these two regions (Rosenhouse-Dantsker et al., 2013). It is possible that this flexibility accounts for a lack of stereospecificity of cholesterol binding to Kir channels that is described above (Singh et al., 2011).

The predicted positions of the cholesterol molecule within both putative cholesterol binding regions that we have identified suggest these regions constitute non-annular cholesterol binding sites. Molecular dynamics simulations provide further support to this hypothesis, demonstrating that these regions are non-annular surfaces that prefer cholesterol to phospholipids (Rosenhouse-Dantsker et al., 2013). Specifically, our simulations showed that for every pose in which cholesterol resided stably for the entire length of the 50 ns molecular dynamics simulation, the bound cholesterol molecule interacted more favorably than a single phospholipid targeting the same residues. Our analysis suggested that this specificity is a result of the matching between the cholesterol molecule and the hydrophobic and aromatic moieties in the two putative cholesterol binding regions. As a result, phospholipids only transiently interact with the residues that form the cholesterol binding surface. According to a contact analysis, during the 50 ns molecular dynamics simulations, there was an average of 35 lipid binding/unbinding events to the two putative cholesterol-specific binding pockets, further supporting the notion that these cholesterol binding regions are non-annular surfaces that are occluded from phospholipid binding.

The strength of the interactions between the cholesterol molecule and each of the two putative cholesterol binding regions can be assessed by calculating the binding enthalpy, a quantitative indicator of changes in the binding energy that depends on two key factors, van-der-Waals interactions between cholesterol and the channel residues, and surface area effects due to expulsion of solvent molecules from hydrophobic surfaces. In addition, the binding affinity and stability of cholesterol to the channel can be assessed by determining the equilibrium free energy of the process that is composed of two thermodynamic components, the binding enthalpy described above and the binding entropy. Combining the contributions of the binding enthalpy and entropy, our calculations indicated that the equilibrium free energy was favorable in region 1 and unfavorable in region 2 (Rosenhouse-Dantsker et al., 2013). Yet, in view of the small absolute value of the free energy in region 1, the binding affinity of cholesterol to this region is expected to be weak. Furthermore, despite the unfavorable equilibrium free energy of cholesterol binding to region 2, we cannot rule out the possibility of weak cholesterol binding to region 2 as well because of its small absolute value and of the typical standard error. Thus, both regions may form cholesterolophilic surfaces with a preference for binding region 1, which is located in the center of the transmembrane domain. Since mutations of both regions abrogated the sensitivity of the channels to cholesterol, region 2 may represent a transient site with weak and possibly short-lived cholesterol binding, which is nevertheless necessary for cholesterol to have an effect on channel function. It is possible that transient binding to the interface region is necessary for cholesterol to access a more stable binding region in the TM domain (Rosenhouse-Dantsker et al., 2013).

#### Implications for channel modulation by cholesterol

Both putative cholesterol binding pockets are located in regions that have been previously shown to play critical roles in the gating mechanism of Kir channels (Figure 5). Cholesterol-binding region 1 at the center of the transmembrane domain overlaps with the hinge region of the inner helix of the channel (Jiang et al., 2002; Jin et al., 2002; Rosenhouse-Dantsker and Logothetis, 2006). The flexibility of this region is required for ensuring the frequent gating of the helix bundle crossing of the channel. It is therefore possible that cholesterol binding may interfere with the hinging motion of the inner helix stabilizing the channel in the closed state. In region 2, cholesterol connects between the slide helix of the N-terminus and the C-linker that connects the C- terminus and the inner transmembrane helix. The interactions between the N- and C- termini have been suggested to provide a tangential force that mechanically gates the channel (Logothetis et al., 2007). Accordingly, interactions between cholesterol and residues located in region 2 may affect the gating mechanism that leads to the opening of the inner helix gate while bending the pore-lining helix at the central glycine hinge. These mechanisms may extend to other ion channels. It has been proposed that a flexible gating hinge in the middle of the inner helix is a common feature in 80% of potassium and cyclic nucleotide-gated channels (Jin et al., 2002). Therefore, if cholesterol binds to the region immediately adjacent to this gating hinge, it may interfere with the hinging motion of the inner helix during channel gating in multiple channels.

## Summary and conclusions

Comparative analysis of sterol effects on different types of ion channels provide growing evidence that multiple channels are regulated primarily by specific sterol-protein interactions rather than by changes in the physical properties of the membrane bilayer. Our earlier studies demonstrated the stereospecificity of cholesterol effects on Kir channels in endothelial cells (Romanenko et al., 2002), which was further confirmed for KirBac channels (Singh et al., 2009) and for purified Kir2.1 channels (D'Avanzo et al., 2010). More recently, cholesterol stereospecificity demonstrated for BK channels (Bukiya et al., 2011) and TRPV1 channels (Picazo-Juarez et al., 2011). Moreover, these observations are consistent with earlier studies in nAChR showing that even though the effect of cholesterol is not stereospecific, it is sensitive to specific sterol substitutions. It is important to note that specific sterol-protein interactions may underlie both the inhibitory (Kir, BK, TRPV1) and the facilitatory effects (nAChR) of cholesterol (Barrantes, 2007; Levitan et al., 2010; Rosenhouse-Dantsker et al., 2012a). In contrast, an example of a mammalian ion channel that has been demonstrated not to be sensitive to specific sterols but rather to lipid packing is VRAC (Romanenko et al., 2004b). Clearly though only a handful of ion channels has been analyzed so far and more work is needed to systematically test different ion channel families. Another major advance in the field is demonstration of binding of cholesterol to an ion channel (KirBac) that provided direct evidence for binding of cholesterol to a purified ion channel.

Significant new insights have also been obtained in elucidating the structural requirements of cholesterol interaction with ion channels. Known cholesterol-binding motifs, CRAC and CARC, have been identified in several types of ion channels (BK, nAChR, and Kir2.1) (Singh et al., 2012; Fantini and Barrantes, 2013; Rosenhouse-Dantsker et al., 2013). Interestingly, it was proposed that among these two motifs, the inverted CRAC motif, CARC may be more energetically beneficial for cholesterol-ion channels interactions (Fantini and Barrantes, 2013). The most extensive site-directed mutagenesis studies so far have been conducted in Kir2.1 channels where numerous mutations in both cytoplasmic (Epshtein et al., 2009; Rosenhouse-Dantsker et al., 2011, 2012b, 2013) and transmembrane domains (Epshtein et al., 2009; Rosenhouse-Dantsker et al., 2011, 2012b, 2013) of the channels provided insight into the molecular mechanisms of cholesterol regulation of these channels. Moreover, recent studies of Kir2.1 revealed two putative cholesterol binding regions at non-annular sites of the transmembrane domain (Epshtein et al., 2009; Rosenhouse-Dantsker et al., 2011, 2012b, 2013). These cholesterol binding sites define three-dimensional binding surfaces that cannot be described by a motif that only includes one simple continuous sequence such as the CRAC or CARC motifs. This notion that cholesterol binds to transmembrane proteins in between several α-helices within the transmembrane domain is further supported by the similar characteristics of the binding sites that were identified in several types of GPCRs such as the β-adrenergic receptor (Cherezov et al., 2007; Hanson et al., 2008) and the dopamine receptor (Penmatsa et al., 2013). Similarly to the case in Kir2.1, also in these two transmembrane proteins primarily hydrophobic residues formed cholesterol binding sites in between α-helices of the transmembrane domain. It is also important to note that while several residues have also been found to decrease or abrogate cholesterol sensitivity of Kir2.1 in the cytosolic domain of the channels, these sites are not expected to constitute cholesterol binding sites. Thus, residues that affect cholesterol sensitivity of the channels may not necessarily be part of a binding site and it is necessary to discriminate between direct and indirect effects of specific mutations. This can be achieved by a combination of computational studies and measuring direct binding.

### Conflict of interest statement

The authors declare that the research was conducted in the absence of any commercial or financial relationships that could be construed as a potential conflict of interest.

## References

[B1] Abi-Char J. Maguy A. Coulombe A. Balse E. Ratajczak P. Samuel J.-L. (2007). Membrane cholesterol modulates Kv1.5 potassium channel distribution and function in rat cardiomyocytes. J. Physiol. 582(Pt 3), 1205 10.1113/jphysiol.2007.134809 17525113 PMC2075263

[B3] Addona G. H. Sandermann H. Jr. Kloczewiak M. A. Husain S. S. Miller K. W. (1998). Where does cholesterol act during activation of the nicotinic acetylcholine receptor? Biochim. Biophys. Acta 1370, 299 10.1016/S0005-2736(97)00280-0 9545586

[B2] Addona G. H. Sandermann H. Jr. Kloczewiak M. A. Miller K. W. (2003). Low chemical specificity of the nicotinic acetylcholine receptor sterol activation site. Biochim. Biophys. Acta 1609, 177–182 10.1016/S0005-2736(02)00685-5 12543379

[B4] Barrantes F. J. (2004). Structural basis for lipid modulation of nicotinic acetylcholine receptor function. Brain Res. Rev. 47, 71–95 10.1016/j.brainresrev.2004.06.008 15572164

[B5] Barrantes F. J. (2007). Cholesterol effects on nicotinic acetylcholine receptor. J. Neurochem. 103, 72 10.1111/j.1471-4159.2007.04719.x 17986142

[B6] Bichet D. Haass F. A. Jan L. Y. (2003). Merging functional studies with structures of inward-rectifier K(+) channels. Nat. Rev. Neurosci. 4, 957–967 10.1038/nrn1244 14618155

[B7] Bolotina V. Omelyanenko V. Heyes B. Ryan U. Bregestovski P. (1989). Variations of membrane cholesterol alter the kinetics of Ca2+-dependent K+ channels and membrane fluidity in vascular smooth muscle cells. Pflugers Arch. 415, 262–268 10.1007/BF00370875 2622758

[B8] Brannigan G. Hénin J. Law R. Eckenhoff R. Klein M. L. (2008). Embedded cholesterol in the nicotinic acetylcholine receptor. Proc. Natl. Acad. Sci. U.S.A. 105, 14418–14423 10.1073/pnas.0803029105 18768796 PMC2567199

[B9] Bukiya A. N. Belani J. D. Rychnovsky S. Dopico A. M. (2011). Specificity of cholesterol and analogs to modulate BK channels points to direct sterol-channel protein interactions. J. Gen. Physiol. 137, 93–110 10.1085/jgp.201010519 PMC3010061 21149543

[B10] Bukiya A. N. Vaithianathan T. Toro L. Dopico A. M. (2008). The second transmembrane domain of the large conductance, voltage- and calcium-gated potassium channel beta(1) subunit is a lithocholate sensor. FEBS Lett. 582, 673 10.1016/j.febslet.2008.01.036 18242174 PMC2665905

[B11] Chang H. M. Reitstetter R. Mason R. P. Gruener R. (1995). Attenuation of channel kinetics and conductance by cholesterol: an interpretation using structural stress as a unifying concept. J. Membr. Biol. 143, 51–63 10.1007/BF00232523 7714888

[B12] Cherezov V. Rosenbaum D. M. Hanson M. A. Rasmussen S. G. Thian F. S. Kobilka T. S. (2007). High-resolution crystal structure of an engineered human β 2-adrenergic G protein-coupled receptor. Science 318, 1258–1265 10.1126/science.1150577 17962520 PMC2583103

[B13] Corbin J. Wang H. H. Blanton M. P. (1998). Identifying the cholesterol binding domain in the nicotinic acetylcholine receptor with [125I]azido-cholesterol. Biochim. Biophys. Acta 1414, 65–74 10.1016/S0005-2736(98)00153-9 9804895

[B14] Criado M. Eibl H. Barrantes F. J. (1982). Effects of lipids on acetylcholine receptor. Essential need of cholesterol for maintenance of agonist-induced state transitions in lipid vesicles. Biochemistry 21, 3622–3629 10.1021/bi00258a015 7115688

[B15] Crowley J. J. Treistman S. N. Dopico A. M. (2003). Cholesterol antagonizes ethanol potentiation of human brain BKCa channels reconstituted into phospholipid bilayers. Mol. Pharmacol. 64, 365–372 10.1124/mol.64.2.365 12869641

[B16] daCosta C. J. B. Dey L. Therien J. P. D. Baenziger J. E. (2013). A distinct mechanism for activating uncoupled nicotinic acetylcholine receptors. Nat. Chem. Biol. 9, 701 10.1038/nchembio.1338 24013278

[B17] daCosta C. J. B. Ogrel A. A. McCardy E. A. Blanton M. P. Baenziger J. E. (2002). Lipid-protein interactions at the nicotinic acetylcholine receptor: a functional coupling between nicotinic receptors and phosphatidic acid-containing lipid bilayers. J. Biol. Chem. 277, 201–208 10.1074/jbc.M108341200 11682482

[B18] Dalziel A. W. Rollins E. S. McNamee M. G. (1980). The effect of cholesterol on agonist-induced flux in reconstituted acetylcholine receptor vesicles. FEBS Lett. 122, 193–196 10.1016/0014-5793(80)80435-2 7202709

[B19] D'Avanzo N. Cheng W. W. L. Doyle D. A. Nichols C. G. (2010). Direct and specific activation of human inward rectifier K+ channels by membrane phosphatidylinositol 4,5-bisphosphate. J. Biol. Chem. 285, 37129–37132 10.1074/jbc.C110.186692 20921230 PMC2988318

[B20] D'Avanzo N. Hyrc K. Enkvetchakul D. Covey D. F. Nichols C. G. (2011). Enantioselective protein-sterol interactions mediate regulation of both prokaryotic and eukaryotic inward rectifier K<sup>+</sup> channels by cholesterol. PLoS ONE 6:e19393 10.1371/journal.pone.0019393 21559361 PMC3084843

[B21] Demel R. A. Bruckdorfer K. R. van Deenen L. L. M. (1972a). The effect of sterol structure on the permeability of liposomes to glucose, glycerol and Rb+. Biochem. Biophys. Acta 255, 321–330 10.1016/0005-2736(72)90031-4 5011000

[B22] Demel R. A. Bruckdorfer K. R. van Deenen L. L. M. (1972b). Structural requirements of sterols for the interaction with lecithin at the air-water interface. Biochem. Biophys. Acta 255, 311–320 10.1016/0005-2736(72)90030-2 5010999

[B23] Deng W. Bukiya A. N. RodrÃguez-Menchaca A. A. Zhang Z. Baumgarten C. M. Logothetis D. E. (2012). Hypercholesterolemia induces up-regulation of KACh cardiac currents via a mechanism independent of phosphatidylinositol 4,5-bisphosphate and Gβ y. J. Biol. Chem. 287, 4925–4935 10.1074/jbc.M111.306134 22174416 PMC3281659

[B24] Dreger M. Krauss M. Herrmann A. Hucho F. (1997). Interactions of the nicotinic acetylcholine receptor transmembrane segments with the lipid bilayer in native receptor-rich membranes. Biochemistry 36, 839 10.1021/bi960666z 9020782

[B25] Ellena J. F. Blazing M. A. McNamee M. G. (1983). Lipid-protein interactions in reconstituted membranes containing acetylcholine receptor. Biochemistry 22, 5523–5535 10.1021/bi00293a012 6317021

[B26] Epand R. M. (2006). Cholesterol and the interaction of proteins with membrane domains. Prog. Lipid Res. 45, 279–294 10.1016/j.plipres.2006.02.001 16574236

[B27] Epshtein Y. Chopra A. Rosenhouse-Dantsker A. Kowalsky G. Logothetis D. E. Levitan I. (2009). Identification of a C-terminus domain critical for the sensitivity of Kir2.1 channels to cholesterol. Proc. Natl. Acad. Sci. U.S.A. 106, 8055–8060 10.1073/pnas.0809847106 19416905 PMC2683107

[B28] Fang Y. Mohler E. R. III. Hsieh E. Osman H. Hashemi S. M. Davies P. F. (2006). Hypercholesterolemia suppresses inwardly rectifying k+ channels in aortic endothelium *in vitro* and *in vivo*. Circ. Res. 98, 1064–1071 10.1161/01.RES.0000218776.87842.43 16556870

[B29] Fantini J. Barrantes F. J. (2013). How cholesterol interacts with membrane proteins: an exploration of cholesterol-binding sites including CRAC, CARC and tilted domains. Front. Physiol. 4:31 10.3389/fphys.2013.00031 23450735 PMC3584320

[B30] Gimpl G. (2010). Cholesterol-protein interaction: methods and cholesterol reporter molecules, in Cholesterol Binding and Cholesterol Transport Proteins, ed Harris J. R. (Dordrecht; Heidelberg; London; New York: Springer), 1–45 10.1007/978-90-481-8622-8_19 20213539

[B31] Gimpl G. Burger K. Fahrenholz F. (1997). Cholesterol as modulator of receptor function. Biochemistry 36, 10959–10974 10.1021/bi963138w 9283088

[B32] Hajdú P. Varga Z. Pieri C. Panyi G. Gáspár R. J. (2003). Cholesterol modifies the gating of Kv1.3 in human T lymphocytes. Pflugers Arch. 445, 674–682 10.1007/s00424-002-0974-y 12632187

[B33] Hamouda A. K. Chiara D. C. Sauls D. Cohen J. B. Blanton M. P. (2006a). Cholesterol interacts with transmembrane alpha-helices M1, M3, and M4 of the Torpedo nicotinic acetylcholine receptor: photolabeling studies using [3H]Azicholesterol. Biochemistry 45, 976 10.1021/bi051978h 16411773 PMC2564873

[B34] Hamouda A. K. Sanghvi M. Sauls D. Machu T. K. Blanton M. P. (2006b). Assessing the lipid requirements of the Torpedo californica Nicotinic acetylcholine receptor. Biochemistry 45, 4327 10.1021/bi052281z 16566607 PMC2527474

[B35] Hanson M. A. Cherezov V. Griffith M. T. Roth C. B. Jaakola V. P. Chien E. Y. T. (2008). A specific cholesterol binding site is established by the 2.8Å structure of the human β-adrenergic receptor. Structure 16, 897–905 10.1016/j.str.2008.05.001 18547522 PMC2601552

[B36] Infante R. E. Abi-Mosleh L. Radhakrishnan A. Dale J. D. Brown M. S. Goldstein J. L. (2008a). Purified NPC1 protein. J. Biol. Chem. 283, 1052–1063 10.1074/jbc.M707943200 17989073

[B37] Infante R. E. Wang M. L. Radhakrishnan A. Kwon H. J. Brown M. S. Goldstein J. L. (2008b). NPC2 facilitates bidirectional transfer of cholesterol between NPC1 and lipid bilayers, a step in cholesterol egress from lysosomes. Proc. Natl. Acad. Sci. U.S.A. 105, 15287–15292 10.1073/pnas.0807328105 18772377 PMC2563079

[B38] Jiang Y. Lee A. Chen J. Cadene M. Chait B. T. MacKinnon R. (2002). The open pore conformation of potassium channels. Nature 417, 523 10.1038/417523a 12037560

[B39] Jin T. Peng L. Mirshahi T. Rohacs T. Chan K. W. Sanchez R. (2002). The beta-gamma subunits of G proteins gate a K+ channel by pivoted bending of a transmembrane segment. Mol. Cell 10, 469 10.1016/S1097-2765(02)00659-7 12408817

[B40] Jones O. T. McNamee M. G. (1988). Annular and nonannular binding sites for cholesterol associated with the nicotinic acetylcholine receptor. Biochemistry 27, 2364–2374 10.1021/bi00407a018 3382628

[B41] Klausen T. K. Hougaard C. Hoffmann E. K. Pedersen S. F. (2006). Cholesterol modulates the volume-regulated anion current in Ehrlich-Lettre ascites cells via effects on Rho and F-actin. Am. J. Physiol. Cell Physiol. 291, C757–C771 10.1152/ajpcell.00029.2006 16687471

[B42] Kuo A. Gulbis J. M. Antcliff J. F. Rahman T. Lowe E. D. Zimmer J. (2003). Crystal structure of the potassium channel KirBac1.1 in the closed state. Science 300, 1922–1926 10.1126/science.1085028 12738871

[B43] Levitan I. (2009). Cholesterol and Kir channels. IUBMB Life 61, 781–790 10.1002/iub.192 19548316 PMC2720429

[B44] Levitan I. Christian A. E. Tulenko T. N. Rothblat G. H. (2000). Membrane cholesterol content modulates activation of volume-regulated anion current in bovine endothelial cells. J. Gen. Physiol. 115, 405–416 10.1085/jgp.115.4.405 10736308 PMC2233759

[B45] Levitan I. Fang Y. Rosenhouse-Dantsker A. Romanenko V. (2010). Cholesterol and ion channels, in Cholesterol Binding and Cholesterol Transport Proteins, ed Harris J. R. (Dordrecht; Heidelberg; London; New York: Springer), 509–549 10.1007/978-90-481-8622-8_1

[B46] Li H. Papadopoulos V. (1998). Peripheral-type benzodiazepine receptor function in cholesterol transport. Identification of a putative cholesterol recognition/interaction amino acid sequence and consensus pattern. Endocrinology 139, 4991–4997 10.1210/endo.139.12.6390 9832438

[B47] Liu M. Huang W. Wu D. Priestley J. V. (2006). TRPV1, but not P2X3, requires cholesterol for its function and membrane expression in rat nociceptors. Eur. J. Neurosci. 24, 1 10.1111/j.1460-9568.2006.04889.x 16800863

[B48] Liu W. Chun E. Thompson A. A. Chubukov P. Xu F. Katritch V. (2012). Structural basis for allosteric regulation of GPCRs by sodium ions. Science 337, 232–236 10.1126/science.1219218 22798613 PMC3399762

[B49] Logothetis D. E. Jin T. Lupyan D. Rosenhouse-Dantsker A. (2007). Phosphoinositide-mediated gating of inwardly rectifying K(+) channels. Pflugers Arch. 455, 83–95 10.1007/s00424-007-0276-5 17520276

[B50] Lundbaek J. A. Andersen O. S. (1999). Spring constants for channel-induced lipid bilayer deformations estimates using gramicidin channels. Biophys. J. 76, 889–895 10.1016/S0006-3495(99)77252-8 9929490 PMC1300090

[B51] Lundbaek J. A. Birn P. Hansen A. J. Andersen O. S. (1996). Membrane stiffness and channel function. Biochemistry 35, 3825–3830 10.1021/bi952250b 8620005

[B52] Lundbaek J. A. Birn P. Hansen A. J. Sogaard R. Nielsen C. Girshman J. (2004). Regulation of sodium channel function by bilayer elasticity: the importance of hydrophobic coupling. effects of micelle-forming amphiphiles and cholesterol. J. Gen. Physiol. 123, 599–621 10.1085/jgp.200308996 15111647 PMC2234500

[B53] Maguy A. Hebert T. E. Nattel S. (2006). Involvement of lipid rafts and caveolae in cardiac ion channel function. Cardiovasc. Res. 69, 798 10.1016/j.cardiores.2005.11.013 16405931

[B54] Manglik A. Kruse A. C. Kobilka T. S. Thian F. S. Mathiesen J. M. Sunahara R. K. (2012). Crystal structure of the micro-opioid receptor bound to a morphinan antagonist. Nature 485, 321–326 10.1038/nature10954 22437502 PMC3523197

[B55] Marsh D. Barrantes F. J. (1978). Immobilized lipid in acetylcholine receptor-rich membranes from *Torpedo marmorata*. Proc. Natl. Acad. Sci. U.S.A. 75, 4329–4333 10.1073/pnas.75.9.4329 212745 PMC336108

[B56] Marsh D. Watts A. Barrantes F. (1981). Phospholipid chain immobilization and steroid rotational immobilization in acetylcholine receptor-rich membranes from *Torpedo-marmorata*. Biochim. Biophys. Acta 645, 97–101 10.1016/0005-2736(81)90516-2 6266478

[B57] Martens J. R. O'Connell K. Tamkun M. (2004). Targeting of ion channels to membrane microdomains: localization of KV channels to lipid rafts. Trends Pharmacol. Sci. 25, 16–21 10.1016/j.tips.2003.11.007 14723974

[B58] Mickus D. E. Levitt D. G. Rychnovsky S. D. (1992). Enantiomeric cholesterol as a probe for ion-channel structure. J. Am. Chem. Soc. 114, 359–360 10.1021/ja00027a055

[B59] Mohler E. R. III. Fang Y. Gusic Shaffer R. Moore J. Wilensky R. L. Parmacek M. (2007). Hypercholesterolemia suppresses Kir channels in porcine bone marrow progenitor cells *in vivo*. Biochem. Biophys. Res. Commun. 358, 317–324 10.1016/j.bbrc.2007.04.138 17482574 PMC2703014

[B60] Murari R. Murari M. P. Baumann W. J. (1986). Sterol orientations in phosphatidylcholine liposomes as determined by deuterium NMR. Biochemistry 25, 1062–1067 10.1021/bi00353a017 3754460

[B61] Penmatsa A. Wang K. H. Gouaux E. (2013). X-ray structure of dopamine transporter elucidates antidepressant mechanism. Nature 503, 85 10.1038/nature12533 24037379 PMC3904663

[B62] Picazo-Juarez G. Romero-Suarez S. Nieto-Posadas A. Llorente I. Jara-Oseguera A. Briggs M. (2011). Identification of a binding motif in the S5 helix that confers cholesterol-sensitivity to TRPV1. J. Biol. Chem. 286, 24966–24976 10.1074/jbc.M111.237537 21555515 PMC3137070

[B63] Popot J. L. Demel R. A. Sobel A. Van Deenen L. L. Changeux J. P. (1978). Interaction of the acetylcholine (nicotinic) receptor protein from *Torpedo marmorata* electric organ with monolayers of pure lipids. Eur. J. Biochem. 85, 27–42 10.1111/j.1432-1033.1978.tb12209.x 639821

[B64] Radhakrishnan A. Sun L.-P. Kwon H. J. Brown M. S. Goldstein J. L. (2004). Direct binding of cholesterol to the purified membrane region of SCAP: mechanism for a sterol-sensing domain. Mol. Cell 15, 259 10.1016/j.molcel.2004.06.019 15260976

[B65] Romanenko V. G. Fang Y. Byfield F. Travis A. J. Vandenberg C. A. Rothblat G. H. (2004a). Cholesterol sensitivity and lipid raft targeting of Kir2.1 channels. Biophys. J. 87, 3850–3861 10.1529/biophysj.104.043273 15465867 PMC1304896

[B67] Romanenko V. G. Rothblat G. H. Levitan I. (2002). Modulation of endothelial inward rectifier K+ current by optical isomers of cholesterol. Biophys. J. 83, 3211–3222 10.1016/S0006-3495(02)75323-X 12496090 PMC1302398

[B66] Romanenko V. G. Rothblat G. H. Levitan I. (2004b). Sensitivity of volume-regulated anion current to cholesterol structural analogues. J. Gen. Physiol. 123, 77–88 10.1085/jgp.200308882 14699079 PMC2217410

[B68] Rosenhouse-Dantsker A. Leal-Pinto E. Logothetis D. E. Levitan I. (2010). Comparative analysis of cholesterol sensitivity of Kir channels: role of the CD loop. Channels 4, 63–66 10.4161/chan.4.1.10366 19923917 PMC2907253

[B69] Rosenhouse-Dantsker A. Levitan I. (2012). Insights into structural determinants of cholesterol sensitivity of Kir channels, in Cholesterol regulation of Ion Channels and Receptors, eds Levitan I. Barrantes F. (Hoboken, NJ: Wiley-Blackwell), 47–67

[B70] Rosenhouse-Dantsker A. Logothetis D. E. (2006). New roles for a key glycine and its neighboring residue in potassium channel gating. Biophys. J. 91, 2860 10.1529/biophysj.105.080242 16877518 PMC1578466

[B71] Rosenhouse-Dantsker A. Logothetis D. E. Levitan I. (2011). Cholesterol sensitivity of KIR2.1 is controlled by a belt of residues around the cytosolic pore. Biophys. J. 100, 381 10.1016/j.bpj.2010.11.086 21244834 PMC3021658

[B72] Rosenhouse-Dantsker A. Mehta D. Levitan I. (2012a). Regulation of ion channels by membrane lipids. Compr. Phsyiol. 2, 31–68 10.1002/cphy.c110001 23728970

[B74] Rosenhouse-Dantsker A. Noskov S. Durdagi S. Logothetis D. E. Levitan I. (2013). Identification of novel cholesterol-binding regions in Kir2 channels. J. Biol. Chem. 288, 31154–31164 10.1074/jbc.M113.496117 24019518 PMC3829427

[B73] Rosenhouse-Dantsker A. Noskov S. Han H. Adney S. K. Tang Q.-Y. RodrÃguez-Menchaca A. A. (2012b). Distant cytosolic residues mediate a two-way molecular switch that controls the modulation of inwardly rectifying potassium (Kir) channels by cholesterol and phosphatidylinositol 4,5-bisphosphate (PI(4,5)P2). J. Biol. Chem. 287, 40266–40278 10.1074/jbc.M111.336339 22995912 PMC3504743

[B75] Rychnovski S. D. Mickus D. E. (1992). Synthesis of ent-cholesterol, the unnatural enantiomer. J. Org. Chem. 57, 2732–2736 10.1021/jo00035a036

[B76] Simmonds A. C. East J. M. Jones O. T. Rooney E. K. McWhirter J. Lee A. G. (1982). Annular and nonannular binding sites on the (Ca2+ +Mg2+)-ATPase. Biochim. Biophys. Acta 693, 398–406 10.1016/0005-2736(82)90447-3 6130787

[B77] Singh A. K. McMillan J. Bukiya A. N. Burton B. Parrill A. L. Dopico A. M. (2012). Multiple cholesterol recognition/interaction amino acid consensus (CRAC) motifs in cytosolic C Tail of Slo1 subunit determine cholesterol sensitivity of Ca2+- and voltage-gated K+ (BK) channels. J. Biol. Chem. 287, 20509–20521 10.1074/jbc.M112.356261 22474334 PMC3370236

[B78] Singh D. K. Rosenhouse-Dantsker A. Nichols C. G. Enkvetchakul D. Levitan I. (2009). Direct regulation of prokaryotic Kir channel by cholesterol. J. Biol. Chem. 284, 30727–30736 10.1074/jbc.M109.011221 19740741 PMC2781626

[B79] Singh D. K. Shentu T.-P. Enkvetchakul D. Levitan I. (2011). Cholesterol regulates prokaryotic Kir channel by direct binding to channel protein. Biochim. Biophys. Acta 1808, 2527 10.1016/j.bbamem.2011.07.006 21798234 PMC3156940

[B80] Sooksawate T. Simmonds M. A. (2001). Effects of membrane cholesterol on the sensitivity of the GABA(A) receptor to GABA in acutely dissociated rat hippocampal neurones. Neuropharmacology 40, 178–184 10.1016/S0028-3908(00)00159-3 11114396

[B81] Szoke E. Borzsei R. Toth D. M. Lengl O. Helyes Z. Sandor Z. (2010). Effect of lipid raft disruption on TRPV1 receptor activation of trigeminal sensory neurons and transfected cell line. Eur. J. Pharmacol. 628, 67 10.1016/j.ejphar.2009.11.052 19958765

[B82] Wacker D. Wang C. Katritch V. Han G. W. Huang X. P. Vardy E. (2013). Structural features for functional selectivity at serotonin receptors. Science 340, 615–619 10.1126/science.1232808 23519215 PMC3644390

[B83] Wada T. Shimono K. Kikukawa T. Hato M. Shinya N. Kim S. Y. (2011). Crystal structure of the eukaryotic light-driven proton-pumping rhodopsin, Acetabularia rhodopsin II, from marine alga. J. Mol. Biol. 411, 986–998 10.1016/j.jmb.2011.06.028 21726566

[B84] Westover E. J. Covey D. F. (2004). The enantiomer of cholesterol. J. Membr. Biol. 202, 61 10.1007/s00232-004-0714-7 15702370

[B85] Xu X. London E. (2000). The effect of sterol structure on membrane lipid domains reveals how cholesterol can induce lipid domain formation. Biochemistry 39, 843–849 10.1021/bi992543v 10653627

[B86] Yeagle P. L. (1985). Cholesterol and the cell membrane. Biochim. Biophys. Acta 822, 267–287 10.1016/0304-4157(85)90011-5 3904832

[B87] Yeagle P. L. (1991). Modulation of membrane function by cholesterol. Biochimie 73, 1303 10.1016/0300-9084(91)90093-G 1664240

[B88] Yeagle P. L. (2013). Non-covalent binding of membrane lipids to membrane proteins. Biochim. Biophys. Acta. [Epub ahead of print]. 10.1016/j.bbamem.2013.11.009 24269542

